# Investigation into patients with suspected adverse reactions to beta-lactams carried out by the allergy unit of Rio de Janeiro General Policlinic

**DOI:** 10.1186/1939-4551-8-S1-A212

**Published:** 2015-04-08

**Authors:** Flavia Carvalho Loyola Loyola

**Affiliations:** 1 Policlinica Geral Do Rio De Janeiro, Brazil

## Background

Due to the high prevalence of patients with suspected adverse reaction (AR) to Beta lactam (BL), this study aims to determine the incidence of hypersensitivity reactions to these drugs, based on ENDA protocol (European Network for Drug Allergy).

## Methods

This study was a retrospective analysis of cases of patients referred to the Allergy Clinic of Rio de Janeiro General Policlinic with records of suggestive of AR to BL, in the period between February 2011 and July 2014. All the patients observed ENDA protocol: specific questionnaire of adverse drug reactions, IgE specific for penicillin G and V, amoxicillin and ampicillin, followed by skin prick tests (SPT) and intradermal (ID) with penicillin and amoxicillin. Finally, the provocation test (DTP) was carried out with Amoxicillin, each step being followed from the previous negative result.

## Results

Forty seven tests were performed, in 35 women (74.4%) with predominant age group between 50 and 70 years (36% of the women) and 12 men (25.5%) without predominant age range from 6 to 62 years old. There were 10 positive tests (21.7%) with 3 positive tests in making the SPT, 5 in ID test with non-immediate reaction and 3 in DTP also with non- immediate reaction. Eighty percent of patients with positive results were women aged between 15 and 62 years (75% over 40 years) and only two male patients aged between 11 and 25 years.

## Conclusions

These results demonstrated the importance of confirming a suspected history of AR to BL through specific tests, since 21.2% of patients have proved to have drug allergy, in contrast with 10% of the international literature. And the negative provocation tests are important because they allow the patient to use the drug tested in the future.

